# Metapaths: similarity search in heterogeneous knowledge graphs via meta-paths

**DOI:** 10.1093/bioinformatics/btad297

**Published:** 2023-05-04

**Authors:** Ayush Noori, Michelle M Li, Amelia L M Tan, Marinka Zitnik

**Affiliations:** Harvard College, Cambridge, MA 02138, United States; Department of Biomedical Informatics, Harvard Medical School, Boston, MA 02115, United States; Department of Biomedical Informatics, Harvard Medical School, Boston, MA 02115, United States; Department of Biomedical Informatics, Harvard Medical School, Boston, MA 02115, United States; Department of Biomedical Informatics, Harvard Medical School, Boston, MA 02115, United States

## Abstract

**Summary:**

Heterogeneous knowledge graphs (KGs) have enabled the modeling of complex systems, from genetic interaction graphs and protein-protein interaction networks to networks representing drugs, diseases, proteins, and side effects. Analytical methods for KGs rely on quantifying similarities between entities, such as nodes, in the graph. However, such methods must consider the diversity of node and edge types contained within the KG via, for example, defined sequences of entity types known as meta-paths. We present metapaths, the first R software package to implement meta-paths and perform meta-path-based similarity search in heterogeneous KGs. The metapaths package offers various built-in similarity metrics for node pair comparison by querying KGs represented as either edge or adjacency lists, as well as auxiliary aggregation methods to measure set-level relationships. Indeed, evaluation of these methods on an open-source biomedical KG recovered meaningful drug and disease-associated relationships, including those in Alzheimer’s disease. The metapaths framework facilitates the scalable and flexible modeling of network similarities in KGs with applications across KG learning.

**Availability and implementation:**

The metapaths R package is available via GitHub at https://github.com/ayushnoori/metapaths and is released under MPL 2.0 (Zenodo DOI: 10.5281/zenodo.7047209). Package documentation and usage examples are available at https://www.ayushnoori.com/metapaths.

## 1 Introduction

Relational data across biological systems—such as the cellular interactome, single cell similarity graphs, gene co-expression networks, and patient interaction networks—can be represented by graph architectures. A simple graph $G=\left(V,\;E\right)$ is defined by a set of nodes V and edges E of a single type. However, real-world networks are often comprised of diverse data modalities; thus, they are poorly modeled by homogenously typed networks. For instance, a homogeneous network is insufficient for modeling the complexities of drug mechanisms and indications. A graph with many types of nodes—such as drugs, diseases, and proteins—that are connected by different relation types—such as “is indicated for,” “is therapeutic target of,” or “physically interacts with”—is necessary. Interconnected objects from various data sources that are represented as a single multigraph with heterogeneous knowledge-informed node and edge types are known as *knowledge graphs* (KGs) (Hogan et al. 2022). Formally, if A is the set of node types with mapping function φ:V→A, the edges E of a KG can be represented as a set of tuples $\left(u,\;v\right)$, where nodes u, v∈V are connected by an edge, and each belongs to a specific node type $\varphi \left(u\right)$ and $\varphi \left(v\right)\in A$.

**Figure 1.**
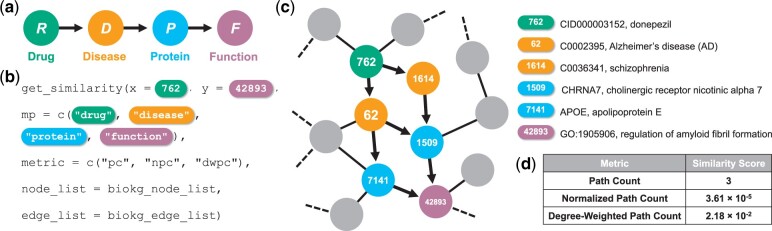
Evaluation of the metapaths package for similarity search in the ogbl-biokg biomedical KG. (a) We query using the *RDPF* (i.e. drug–disease–protein–function) meta-path. (b) The function call used to calculate meta-path-based similarity scores is shown. (c) The meta-path traversal function identifies three paths following the specified meta-path that connect donepezil—a drug used to treat Alzheimer’s disease (AD)—with the regulation of amyloid fibril formation pathway, which is implicated in AD (Supplementary data). (d) The computed similarity scores using Path Count, Normalized Path Count, and Degree-Weighted Path Count metrics are shown

Relationships between entities in KGs are modeled by network similarities quantified using sequences of nodes—or linkage paths—in the network. However, meaningful similarity search methods on KGs must account for the diverse types in these walks. For example, consider a KG of the biological interactome with the following node types: A={disease (*D*), drug (*R*), protein (*P*), protein function (*F*), side effect (*S*)}. Classic random walk-based similarity metrics would not differentiate between the following paths of length three: *RDP* (i.e. drug, disease, protein) and *RSP* (i.e. drug, side effect, protein), even though the former considers disease-mediated associations while the latter considers mechanisms of side effects (Fig. 1).

To distinguish between such paths of identical lengths, we leverage meta-paths, a general graph-theoretic approach for flexible similarity search in large networks. Meta-paths are sequences of node types which define a walk from an origin node to a destination node (Sun et al. 2011b). Note that edge types may also be specified in the walk; here, we do not consider such meta-paths. Fundamentally, the similarity between an origin node and a destination node is measured by the number of meta-paths that exist between them. While meta-paths are frequently used in biomedical network analysis (e.g. Fu et al. 2016; Himmelstein et al. 2017; Zhang et al. 2020), there is currently no package available in R that offers a wide range of support for meta-paths.

Informative meta-paths in KGs are often engineered by hand based on domain knowledge or expertise (e.g. the meta-path *DRS* is clinically meaningful, since it describes associations between a disease and the side effects of its treatments, whereas the meta-path *PSF* would not be). Alternatively, optimal meta-paths can be discovered in an unsupervised fashion by feature selection metrics [e.g. maximal spanning tree (Wang et al. 2018), Laplacian score, or ranking based on meta-path frequency or uniqueness (Zhu et al. 2018)] after enumerating meta-paths from a sampled subset of nodes $V_{M}\subset V$, multi-hop reasoning with reinforcement learning (Wan et al. 2020), or graph attention applied on vector embedded meta-path instance sets (Li et al. 2021), among other approaches.

Once informative meta-paths for a given KG have been identified, these meta-paths define the semantics of the relationships between nodes in the KG, thereby enabling heterogeneous graph convolutional (Zhang et al. 2019) and graph attention (Wang et al. 2019) networks for downstream machine learning analyses such as link prediction (Himmelstein et al. 2017), node classification (Wang et al. 2021), and subgraph prediction (Alsentzer et al. 2020); additional downstream applications are discussed in the Supplementary data. Although various algorithms exist to model meta-path-based node similarities in a KG, a unifying framework is lacking to compute and compare these similarity scores. Here, we introduce metapaths which, to the best of our knowledge, is the first software package in the R ecosystem to implement meta-paths. The metapaths package enables the computation of meta-path-based similarity search in heterogeneous KGs.

## 2 Implementation and evaluation

The primitives of the metapaths package identify the neighbors of a specified node with a given type by querying either an edge list or, for efficiency, an adjacency list precomputed from an edge list. The meta-path traversal function accepts an origin node, a destination node, and a specified meta-path; then, via the neighbor identification functions, it starts at the origin node and recursively expounds the sequence of node types until the destination node is reached. The resulting paths are used to compute meta-path-based similarity scores using various available similarity metrics, including the natively supported Path Count, Normalized Path Count, Degree-Weighted Path Count, and PathSim (Supplementary data) (Sun et al. 2011a,b; Himmelstein and Baranzini 2015). Users may also use the framework provided by the metapaths package to define and test custom similarity metrics of their choosing or evaluate the similarity between two sets of nodes via auxiliary aggregation functions.

To validate the metapaths package, we demonstrate that the package similarity functions report higher connectivity in the ogbl-biokg (BioKG) biomedical KG (Hu et al. 2020) between Alzheimer’s disease (AD)-related drugs (e.g. donepezil, memantine, and galantamine) and AD-related pathways (Supplementary Table S1); this increased connectivity also holds at both the node pair and node set level (Fig. 1 and Supplementary data). Finally, by testing metapaths functions on randomly sampled BioKG subgraphs of increasing size, we demonstrate that the performance of the metapaths package scales well with input size (Supplementary Fig. S1).

## 3 Conclusion

The metapaths R software package facilitates the scalable and flexible modeling of network similarities in KGs. Relationships between individual nodes in a KG can be quantified using built-in or user-defined similarity metrics; such metrics can also be applied to model set-level relationships via aggregation methods. Evaluation on AD-related pathways in BioKG recovers meaningful drug and disease-associated relationships as quantified by high similarity scores. The applications of such similarity search in KGs extend across KG learning.

## Supplementary Material

btad297_Supplementary_Data

## Data Availability

The metapaths R package is available via GitHub at https://github.com/ayushnoori/metapaths and is released under MPL 2.0 (Zenodo DOI: 10.5281/zenodo.7047209). Package documentation and usage examples are available at https://www.ayushnoori.com/metapaths. The ogbl-biokg and ogbn-arxiv datasets are publicly available from the Open Graph Benchmark at https://ogb.stanford.edu.
