# Supplementary material for: Metapaths: similarity search in heterogeneous knowledge graphs via meta-paths
Source: Bioinformatics. 2023 May 4;39(5):btad297. doi: 10.1093/bioinformatics/btad297 (PMC10209523; doi:10.1093/bioinformatics/btad297)
Supplement: btad297_Supplementary_Data [file btad297_supplementary_data.zip › Noori et al. 2022 - Supplementary Information.pdf]

Supplementary Information

metapaths: similarity search in heterogeneous knowledge graphs via meta paths

Ayush Noori[1,2], Michelle M. Li[2,†], Amelia L. M. Tan[2,†], and Marinka Zitnik[2,*]

[1]Harvard College, Cambridge, MA 02138, USA

[2]Department of Biomedical Informatics, Harvard Medical School, Boston, MA 02115, USA

[†]Authors contributed equally.

*To whom correspondence should be addressed.

1 Package Implementation

The primitives of the `metapaths` package identify the neighbors of a specified node with a given type by querying either an edge list or, for efficiency, an adjacency list precomputed from the edge list. The meta path traversal function accepts an origin node, a destination node, and a specified meta path; then, via the neighbor identification functions, it starts at the origin node and recursively expounds the sequence of node types until the destination node is reached. The resulting paths are used to compute meta-path-based similarity scores.

1.1 Similarity Metrics

Various metrics exist to compute node similarities from meta paths in a knowledge graph (KG). Given an origin node, a destination node, and a specified meta path, as well as a set of desired similarity metrics, the `metapaths` package will compute the various similarity scores between the two nodes based on the provided metrics. The following metrics are natively supported:

1. Path Count: the number of paths of a specified meta path between the origin node and the destination node.
2. Normalized Path Count: the path count normalized by the individual connectivities of the origin and destination nodes in the KG to counteract the overrepresentation of non-informative paths traversing high-degree nodes. (Sun, Barber, *et al.*, 2011).
3. Degree-Weighted Path Count: the path count where each path is normalized by the path-degree-product (PDP; i.e., for a given path, the product of the degrees of each node along the path, where the degrees are exponentiated to some damping exponent $-w$ such that $w \leq 0$) to individually downweigh paths that traverse high-degree nodes (Himmelstein and Baranzini, 2015). By default, $w = 0.4$.
4. PathSim: a metric defined on symmetric meta paths that normalizes the path count by the visibility; i.e., for both the origin and destination nodes, the number of paths of the specified meta path between themselves (Sun, Han, *et al.*, 2011).

Users may also use the framework provided by the `metapaths` package to define and test custom similarity metrics of their choosing.

1.2 Aggregation and Comparison

The `metapaths` package also offers the functionality for users to evaluate the similarity between two sets of nodes: for example, in the KG of the interactome, two sets of protein nodes, where each set represents genes in different biological pathways. This comparison is performed by aggregating similarity scores across each set. By default, the maximum similarity scores per node in the origin set are averaged (Guney *et al.*, 2016); however, other aggregation approaches are available and users may also use the `metapaths` package to test their own custom methods. In addition, different metrics and meta paths can be simultaneously evaluated in a single function call. The requisite steps to define custom similarity metrics and aggregation methods are detailed at https://github.com/ayush-noori/metapaths/blob/master/README.md.

2 Evaluation on a Biomedical KG

We evaluate the `metapaths` package on the `ogbl-biokg` (BioKG) dataset from the Open Graph Benchmark (Hu *et al.*, 2020), a biomedical KG that describes relationships between the following node types: disease (*D*, 10,687 nodes), drug (*R*, 10,533 nodes), protein (*P*, 17,499 nodes), protein function (*F*, 45,085 nodes), and side effect (*S*, 9,969 nodes).

We consider the nonsymmetric meta path *RDPF* (i.e., drug-disease-protein-function, see Figure 1A) to investigate the relationships between drugs of interest and biological processes, molecular functions, or cellular components in the Gene Ontology database (Gene Ontology Consortium, 2021; Ashburner *et al.*, 2000). Specifically, we first query the relationship between donepezil – a centrally acting reversible acetylcholinesterase inhibitor frequently prescribed to enhance cognitive function in patients with Alzheimer's disease (AD) – with the regulation of amyloid fibril formation (RAFF) pathway (Figure 1B). Aggregation of amyloid fibrils is associated with AD; thus, we attempt to recover the AD-mediated relation between donepezil and the RAFF pathway using the `metapaths` package. The meta path traversal function shown in Figure 1B identifies three paths following the specified meta path that connect donepezil to the RAFF pathway via both Alzheimer's disease and schizophrenia (Figure 1C), with the similarity scores shown in Figure 1D. The intermediate nodes in these paths are pertinent to AD: the ε4 allele of the apolipoprotein

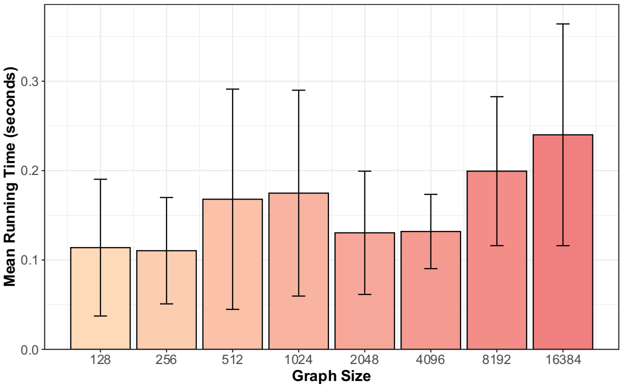

Supplementary Figure 1. Performance of the `get_similarity()` function for similarity search on `ogbl-biokg` subgraphs of various sizes. For each graph size, 10 subgraphs were randomly sampled from BioKG. Then, within each subgraph, the execution time of `get_similarity()` for Degree-Weighted Path Count (DWPC) computation with a random origin node, random destination node, and random meta path of length three connecting the two was measured 10 times and averaged to compute the mean running time. Error bars represent mean ± SD.

E (*APOE*) gene is the most significant genetic risk factor for sporadic AD (Serrano-Pozo *et al.*, 2021), while polymorphisms in *CHRNA7*, which encodes the α7 subunit of the nicotinic acetylcholine receptor, are associated with the response to cholinesterase inhibitors like donepezil in AD (Weng *et al.*, 2013). Encouragingly, the similarity scores between donepezil and other AD related pathways (e.g., "amyloid beta clearance," path count: 5, normalized path count: 6.02×10^{-5}, degree-weighted path count: 1.44×10^{-2}) are also high, whereas the scores between donepezil and 100 randomly sampled, unrelated pathways are zero for 84 out of the 100 comparisons across all three similarity metrics tested (path count: 0, 1.19 ± 4.95, normalized path count: 0, $1.39 \times 10^{-5} \pm 5.68 \times 10^{-5}$, degree-weighted path count: 0, $1.46 \times 10^{-3} \pm 4.84 \times 10^{-3}$; median, mean ± SD). In sum, using the `metapaths` package, we are able to quantify the higher connectivity in BioKG between AD-related drugs and AD-related pathways.

Next, to demonstrate set-to-set comparison with `metapaths`, we compute similarity scores between a cassette of AD-related drugs (namely, donepezil, memantine, and galantamine) and a set of six AD-related pathways as well as a set of six randomly sampled pathways (Supplementary Table 1). Using the default maximum aggregation method, the similarity scores between the drug cassette and the AD-related pathways are as follows: path count: 5, normalized path count: 6.02×10^{-5}, degree-weighted path count: 1.44×10^{-2}. Conversely, the scores between the drugs and the set of randomly sampled pathways are zero across all three metrics. Thus, the increased connectivity between AD-related drugs and pathways holds at the node set level.

In addition to biomedical KGs, the `metapaths` package can compute meta-path-based similarity scores on other heterogeneous KGs as well. For example, we evaluate the `metapaths` package on the `ogbn-arxiv` KG, also available from the Open Graph Benchmark (Hu *et al.*, 2020). This KG represents the citation network between computer science (CS) papers in arXiv indexed by the Microsoft Academic Graph (MAG) (Wang *et al.*, 2020). Each node in this KG is an arXiv CS paper, and directed edges indicate that the origin paper cites the destination paper. Nodes in this dataset can have one of 40 types, where each type represents a subject area of arXiv CS papers (*e.g.*, cs.AI, cs.LG, or cs.OS), an eight-fold increase compared to the five node types in the `ogbl-biokg` dataset. Code demonstrating the functionality of the `metapaths` package on the `ogbl-biokg` and `ogbn-arxiv` datasets is available at https://www.ayushnoori.com/metapaths.

3 Performance Evaluation

We also investigated how the performance of key functions of the `metapaths` package scales with input size. To do so, we first generated subgraphs of BioKG of various sizes by randomly sampling a root node, then successively adding neighbors to augment the subgraph until the desired number of nodes was reached. We produced subgraphs of sizes 2^7 through 2^{14}; for each graph size, 10 subgraphs were randomly sampled. Then, within each subgraph, the execution time of the `get_similarity()` function for Degree-Weighted Path Count (DWPC) computation with a random origin node, random destination node, and random meta path of length three connecting the two was measured 10 times and averaged to compute the mean running time. The results demonstrate that the performance of the core function of the `metapaths` package scales well across BioKG subgraphs that differ in size by seven orders of base-2 magnitude (Supplementary Figure 1).

4 Downstream Applications

The `metapaths` package is designed as a general-purpose toolkit for meta-path-based similarity score computation; these similarity scores can be provided as input to various downstream tasks, including link prediction, node classification, and subgraph prediction, as discussed in the main text. In the biomedical domain, meta paths are particularly pertinent to representation learning and interaction prediction tasks in heterogeneous biomedical KGs. We highlight some examples below:

- Wang *et al.* use a meta path-driven deep Transformer encoder to learn node representations for disease-gene association prediction (Wang *et al.*, 2022).
- Yao *et al.* apply a meta-path-based feature learning module – including meta-path-based information propagation and a meta-path-based attention mechanism – to learn node representations for drug-side effect association prediction (Yao *et al.*, 2022).
- Tanvir *et al.* use meta-path-based topological features – including Path Count and Normalized Path Count, which are natively supported by the `metapaths` package – to extract semantic relationships for drug-drug interaction prediction (Tanvir *et al.*, 2021).
- Deng *et al.* learn meta-path-based feature vectors of RNA-disease pairs for the prediction of circular RNA (circRNA)-disease associations (Deng *et al.*, 2020) and long non-coding RNA (lncRNA)-disease associations (Deng *et al.*, 2021).
- Jin *et al.* employ meta-path-based random walks to learn microRNA-mediated disease comorbidities and identify shared underlying disease pathways (Jin *et al.*, 2019).
- Tian *et al.* predict drug-disease associations for drug repurposing using the HeteSim meta-path-based similarity score (Tian *et al.*, 2018; Shi *et al.*, 2014).
- Himmelstein *et al.* leverage meta paths for drug repurposing (Himmelstein *et al.*, 2017) and for multiscale network integration – across diverse diseases, genes, gene sets, tissues, and pathophysiologies – to enable disease gene prioritization (Himmelstein and Baranzini, 2015).

These and other examples demonstrate the broad utility of meta paths for biomedical applications, suggesting that the `metapaths` package will be a valuable tool for meta-path-based biomedical learning.

Code and Data Availability

The `metapaths` R package is freely available under MPL 2.0 via GitHub at https://github.com/ayushnoori/metapaths (Zenodo DOI: 10.5281/zenodo.7047209).

Documentation and examples are at https://www.ayushnoori.com/metapaths. The `ogbl-biokg` and `ogbn-arxiv` datasets are publicly available from the Open Graph Benchmark at https://ogb.stanford.edu.

Supplementary References

Ashburner, M. *et al.* (2000) Gene Ontology: tool for the unification of biology. *Nat Genet*, **25**, 25–29.

Deng, L. *et al.* (2021) LDAH2V: Exploring Meta-Paths Across Multiple Networks for lncRNA-Disease Association Prediction. *IEEE/ACM Transactions on Computational Biology and Bioinformatics*, **18**, 1572–1581.

Deng, L. *et al.* (2020) Predicting circRNA-disease associations using meta path-based representation learning on heterogenous network. In, *2020 IEEE International Conference on Bioinformatics and Biomedicine (BIBM)*, pp. 5–10.

Gene Ontology Consortium (2021) The Gene Ontology resource: enriching a GOld mine. *Nucleic Acids Res*, **49**, D325–D334.

Guney, E. *et al.* (2016) Network-based in silico drug efficacy screening. *Nat Commun*, **7**, 10331.

Himmelstein, D.S. *et al.* (2017) Systematic integration of biomedical knowledge prioritizes drugs for repurposing. *eLife*, **6**, e26726.

Himmelstein, D.S. and Baranzini, S.E. (2015) Heterogeneous Network Edge Prediction: A Data Integration Approach to Prioritize Disease-Associated Genes. *PLOS Computational Biology*, **11**, e1004259.

Hu, W. *et al.* (2020) Open Graph Benchmark: Datasets for Machine Learning on Graphs. In, *Advances in Neural Information Processing Systems*. Curran Associates, Inc., pp. 22118–22133.

Jin, S. *et al.* (2019) A network-based approach to uncover microRNA-mediated disease comorbidities and potential pathobiological implications. *npj Syst Biol Appl*, **5**, 1–11.

Serrano-Pozo, A. *et al.* (2021) APOE and Alzheimer's disease: advances in genetics, pathophysiology, and therapeutic approaches. *Lancet Neurol*, **20**, 68–80.

Shi, C. *et al.* (2014) HeteSim: A General Framework for Relevance Measure in Heterogeneous Networks. *IEEE Transactions on Knowledge and Data Engineering*, **26**, 2479–2492.

Sun, Y., Barber, R., *et al.* (2011) Co-author Relationship Prediction in Heterogeneous Bibliographic Networks. In, *2011 International Conference on Advances in Social Networks Analysis and Mining*, pp. 121–128.

Sun, Y., Han, J., *et al.* (2011) PathSim: meta path-based top-K similarity search in heterogeneous information networks. *Proc. VLDB Endow.*, **4**, 992–1003.

Tanvir, F. *et al.* (2021) Predicting Drug-Drug Interactions Using Meta-path Based Similarities. In, *2021 IEEE Conference on Computational Intelligence in Bioinformatics and Computational Biology (CIBCB)*, pp. 1–8.

Tian, Z. *et al.* (2018) Computational drug repositioning using meta-path-based semantic network analysis. *BMC Systems Biology*, **12**, 134.

Wang, H. *et al.* (2022) deepDGA: Biomedical Heterogeneous Network-based Deep Learning Framework for Disease-Gene Association Predictions. In, *2022 IEEE International Conference on Bioinformatics and Biomedicine (BIBM)*, pp. 601–606.

Wang, K. *et al.* (2020) Microsoft Academic Graph: When experts are not enough. *Quantitative Science Studies*, **1**, 396–413.

Weng, P.-H. *et al.* (2013) CHRNA7 polymorphisms and response to cholinesterase inhibitors in Alzheimer's disease. *PLoS One*, **8**, e84059.

Yao, W. *et al.* (2022) MPGNN-DSA: A Meta-path-based Graph Neural Network for drug-side effect association prediction. In, *2022 IEEE International Conference on Bioinformatics and Biomedicine (BIBM)*, pp. 627–632.
